# Evidence for a Role of Orexin/Hypocretin System in Vestibular Lesion-Induced Locomotor Abnormalities in Rats

**DOI:** 10.3389/fnins.2016.00355

**Published:** 2016-07-26

**Authors:** Leilei Pan, Ruirui Qi, Junqin Wang, Wei Zhou, Jiluo Liu, Yiling Cai

**Affiliations:** Department of Nautical Injury Prevention, Faculty of Navy Medicine, Second Military Medical UniversityShanghai, China

**Keywords:** vestibular lesion, orexin, hypothalamus, locomotor activity, exploratory behavior

## Abstract

Vestibular damage can induce locomotor abnormalities in both animals and humans. Rodents with bilateral vestibular loss showed vestibular deficits syndrome such as circling, opisthotonus as well as locomotor and exploratory hyperactivity. Previous studies have investigated the changes in the dopamine system after vestibular loss, but the results are inconsistent and inconclusive. Numerous evidences indicate that the orexin system is implicated in central motor control. We hypothesized that orexin may be potentially involved in vestibular loss-induced motor disorders. In this study, we examined the effects of arsanilate- or 3,3′-iminodipropionitrile (IDPN)-induced vestibular lesion (AVL or IVL) on the orexin-A (OXA) labeling in rat hypothalamus using immunohistochemistry. The vestibular lesion-induced locomotor abnormalities were recorded and verified using a histamine H4 receptor antagonist JNJ7777120 (20 mg/kg, i.p.). The effects of the orexin receptor type 1 antagonist SB334867 (16 μg, i.c.v.) on these behavior responses were also investigated. At 72 h post-AVL and IVL, animals exhibited vestibular deficit syndrome and locomotor hyperactivity in the home cages. These responses were significantly alleviated by JNJ7777120 which also eliminated AVL-induced increases in exploratory behavior in an open field. The numbers of OXA-labeled neurons in the hypothalamus were significantly increased in the AVL animals at 72 h post-AVL and in the IVL animals at 24, 48, and 72 h post-IVL. SB334867 significantly attenuated the vestibular deficit syndrome and locomotor hyperactivity at 72 h post-AVL and IVL. It also decreased exploratory behavior in the AVL animals. These results suggested that the alteration of OXA expression might contribute to locomotor abnormalities after acute vestibular lesion. The orexin receptors might be the potential therapeutic targets for vestibular disorders.

## Introduction

Peripheral vestibular system located in the inner ear conveys information about the body motion and the gravity (Wiest, 2015). Previous studies confirmed that bilateral vestibular loss produces remarkable behavior abnormalities (circling, retropulsion, opisthotonus, and moderate ataxia) and hyperactivity in the animals receiving mechanical or chemical labyrinthectomy and in the mice with genetic deficiency in the vestibular endorgans (Llorens and Rodríguez-Farré, 1997; Kaiser et al., 2001; Schirmer et al., 2007a; Goddard et al., 2008; Vignaux et al., 2012). These behavior responses occur spontaneously or in response to stress such as placing the animal in a new environment (Lindemann et al., 2001). Animals with the deficits in the inner ear also exhibited marked vestibular dysfunction during the air-righting reflex test and the tail-hanging test (Hunt et al., 1987; Ossenkopp et al., 1990). Recently, it was reported that the vestibular deficits induced by systemic administration of 3,3′-iminodipropionitrile (IDPN) or by intra-tympanic injections of kainate were improved by the histamine H_4_ receptor (H_4_R) antagonists which exhibited pronounced inhibitory effects on the vestibular neuron activity (Desmadryl et al., 2012; Wersinger et al., 2013). This indicates that the vestibular damage is responsible for the behavior abnormalities such as circling, opisthotonus, and locomotor hyperactivity.

Although there was evidence that vestibular loss may potentially influence the activity of the forebrain dopamine systems, the results that were derived from different types of vestibular deficits were inconsistent (Löscher, 2010). Pharmacological studies showed that the dopamine D_2_ receptor antagonist eliminated the hyperactivity and circling behaviors in the ci2 mutant rats with vestibular deficits but not in animals receiving surgical vestibular deafferentation (Schirmer et al., 2007b; Stiles et al., 2012). Histochemical examination of the striatum and the substantia nigra pars compacta failed to disclose any abnormalities in the density of the dopaminergic neurons in streptomycin-treated LEW/Ztm rats, whereas a significant asymmetry in the density of the dopaminergic neurons was determined in the ventral tegmental area in the ci2 mutant rats (Schirmer et al., 2007a). These results strongly suggested that the vestibular loss-related motor disorders cannot be solely explained by the functional alterations in the dopaminergic system (Palomero-Gallagher et al., 2008, 2010).

Recently, the orexin (hypocretin) neuropeptide producing neurons which are located in the hypothalamus including the lateral hypothalamic area (LHA), the perifornical area (PFA), the dorsomedial hypothalamus (DMH), and the posterior hypothalamus were found to play critical roles in regulating sleep and wakefulness (Sinton, 2011). Numerous evidences indicate that the orexin system also plays key roles in central motor control (Hu et al., 2015). Orexin-A (OXA) that was administrated into the hypothalamic paraventricular nucleus dose-dependently increased the spontaneous physical activity in rats, while the orexin receptor type 1 (OX_1_R) antagonist SB334867 significantly attenuated such effects (Kiwaki et al., 2004). The increases in the locomotor activity were also observed after the injection of the orexin peptides into the rostral lateral hypothalamic area, the nucleus accumbens, the locus coeruleus, the dorsal raphe nucleus, the tuberomammillary nucleus, or the substantia nigra (Zink et al., 2014). Moreover, the elevated orexin receptor expression levels were found to be associated with higher activity in the obesity-resistant rats than in the obese rats (Teske et al., 2006, 2013). OXA dose-dependently potentiated the contraversive pivoting that was induced by the activation of the dopamine D_1_ and D_2_ receptor in the nucleus accumbens shell, whereas SB334867 did not affect the pivoting (Kotani et al., 2008). In addition, anatomical studies have demonstrated that the vestibular nucleus complex has reciprocal connections with the LHA (Matsuyama et al., 1996; Horowitz et al., 2005; Ciriello and Caverson, 2014). The brainstem regions which receive the direct innervations from the vestibular nucleus such as the dorsal raphe nucleus, the lateral parabrachial nucleus and the locus coeruleus also contain considerable efferent neurons that directly project to the LHA region (Yoshida et al., 2006). Based on these observations, we hypothesized that the orexin system may be potentially involved in the motor disorders that are induced by vestibular dysfunction.

The present study observed the alteration of OXA labeling in the hypothalamus and tested the behavior abnormalities that were induced by two different approaches of chemical vestibular lesion (VL) in rats. The chemical vestibular lesions were induced by the systemic injection of IDPN or the intra-tympanic injection of sodium arsanilate. These chemicals can cause lesions in the vestibular hair cells and sensory epithelia as previously described (Desmadryl et al., 2012; Martin et al., 2015). Vestibular deficit syndrome (circling, retropulsion and head bobbing as well as contact inhibition of the righting reflex and abnormalities in tail-hanging reflex and air-righting reflex), spontaneous locomotor activity in the home cages and exploratory behavior in a new environment (open field) were observed after the vestibular lesions. The VL-related behavior responses were verified via the application of a selective non-imidazole H_4_R antagonist JNJ7777120 which has been found to be a pronounced inhibitor on the activity of the vestibular neurons at a dose of 20 mg/kg body weight in rats (Desmadryl et al., 2012). The efficacy of the OX_1_R inhibitor SB334867 on the VL-induced behavior abnormalities was also investigated.

## Materials and methods

### Animals and ethics

Adult male Sprague–Dawley rats weighing 250–300 g were purchased from the Laboratory Animal Center in Shanghai. The animals were individually housed under a 12 h light: 12 h dark cycle (temperature: 22 ± 2°C and lighting: 8:00–20:00) with free access to food and water. All animals were acclimated to the lab environment with the temperature maintained at 22°C for 2 weeks before the initiation of the experiment. All animal protocols and procedures complied with the Guide for the Care and Use of Laboratory Animals (National Research Council (US) Institute for Laboratory Animal Research, 1996) and were approved by the Ethics Committee for Animal Experimentation of the Second Military Medical University (Shanghai, PR China). Efforts were made to minimize the number of animals used and their suffering in each experiment. The implantation surgeries were performed under sodium pentobarbital (40 mg/kg, i.p.) anesthesia. The animals were allowed a 7-day recovery after the surgery. The intra-tympanic injections were conducted under volatile anesthesia (2% isoflurane) in oxygen (flow rate of 2 L/min). All animals received antibiotics penicillin (400000 U/kg, i.p.) and analgesics ibuprofen (30 mg/kg, in the drinking water) once a day for 3 days after the implantation surgery and the intra-tympanic injection.

### Vestibular lesion procedures

Two different models of chemical vestibular lesion were produced in this study. In the arsanilate**-**induced VL (AVL) model, vestibular deficits were elicited by the intra-tympanic injection of a single dose (50–100 μl) of sodium arsanilate (30% w/v) bilaterally in rats. The sham control (SHAM) animals received the intra-tympanic injections of saline (0.9% w/v) following the same procedures. In the IDPN**-**induced VL (IVL) model, vestibular deficits were produced by the intraperitoneal (i.p.) injection of a single dose of IDPN (900 mg/kg body weight) in rats. The saline control (SAL) animals received the i.p. injection of 1 ml saline solution (0.9% w/v) instead. All these operations were performed between 4:00 and 6:00 p.m. All animals were allowed a 2-h recovery before further experiments.

### Experimental design and grouping

#### Experiment 1

In this experiment, OXA labeling in the hypothalamus was observed in the VL animals. Seventy-two rats were used and randomly divided into the following groups: the AVL or the IVL group and the corresponding SHAM or SAL group (*n* = 18 in each group). Animals in each group were evenly assigned into three subgroups after the recovery from the VL or control treatment (*n* = 6 in each subgroup). They received the behavior tests at 24, 48, or 72 h post-VL, respectively, and were killed for OXA immunostaining immediately after the tests.

#### Experiment 2

The effects of the H_4_R antagonist JNJ7777120 on the VL-induced behavioral responses were observed in this part. Sixty-four animals were used and randomly divided into eight groups: two AVL or two IVL groups and corresponding two SHAM or two SAL groups. Animals in the two groups in pairs received an i.p. injection of either 1 ml JNJ7777120 (JNJ, 20 mg/kg body weight) or 1 ml vehicle (Veh) solution (dimethyl sulfoxide, DMSO) at 72 h after the VL or control treatment (*n* = 8 in each group). All rats received behavioral test 30 min after the drug injections.

#### Experiment 3

The effects of the intracerebroventricular (i.c.v.) injection of the OX_1_R antagonist SB334867 (SB) on the VL-induced behavior responses were examined. The animal groupings and the experimental procedures in this experiment were the same as the Experiment 2, instead that the animals were injected with 4 μl SB334867 (16 μg) or 4 μl Veh (i.c.v.) 30 min before the behavior tests according to the pilot studies. At the end, 6 ml of 2% pontamine sky blue solution was administrated to mark the injection sites after the termination of the animals with overdose pentobarbitone (100 mg/kg, i.p.). Only those animals with the blue staining in the lateral and fourth ventricles were included in the further analyses.

### Surgery procedures

#### Intracerebroventricular injection

A 23-gauge stainless steel guide cannulae (RWD Life Science, Shenzhen, China) was implanted into the right lateral ventricle (0.9 mm caudal to the Bregma, 1.5 mm lateral to the midline, and 3.5 mm below the dura). During drug administration, the guide cannulae were fitted with a 30-gauge stainless steel injection needle that extended 0.5 mm beyond the tip of the guide cannulae. The drug solution was injected by pressure over a period of 5 s.

#### Activity sensor implantation

The implantation surgery was carried out according to the manufacturer's instructions (DSI, St. Paul, USA). An activity sensor (TA10TA-F40) was implanted intra-abdominally after midline laparotomy and then the abdominal muscles and skins were sutured under anesthesia.

### Behavior test

#### Vestibular deficit test

The vestibular deficit syndrome was evaluated by observing the behavior responses including the behavior abnormalities and the vestibular reflex dysfunctions described previously (Vignaux et al., 2012). During the behavior abnormality test, rats were placed for 1 min on a table top, and the experimenter rated the animals from 0 to 4 for circling, retropulsion, and abnormal head movements. Circling was defined as stereotyped circling movement. Retropulsion consisted of backward displacement of the animal. Head bobbing consisted of intermittent extreme backward extension of the neck. During vestibular reflex test, tail-hanging reflex, contact inhibition of the righting reflex, and the air-righting reflex test were examined. For the tail-hanging reflex test, animals were lifted by the tail. Normal rats exhibit a “landing” response consisting of forelimb extension, while rats with impaired vestibular function bent ventrally, sometimes “crawling” up toward their tails, and thus tending to occipital landing. For the contact inhibition of the righting reflex test, rats were placed supine on a horizontal surface, and a metal bar grid was lightly placed in contact with the soles of the animals' feet. Healthy rats quickly right themselves, whereas the vestibular-deficient rats lie on their back, with their feet up, and “walk” with respect to the ventral surface. For the air-righting reflex test, the animals were held supine and dropped from a height of 40 cm onto a foam cushion. A vestibular deficit score was obtained by adding up the scores for all of the behavior responses and expressed as a percentage of the maximal score of 24 (Hunt et al., 1987; Ossenkopp et al., 1990; Vignaux et al., 2012).

#### Open field test

The exploratory behavior was measured using an open field test system (RD1112-IFO-R-4, Mobiledatum, Shanghai, China) immediately after the vestibular deficit test. The apparatus consisted of a dark 40 × 40 × 45 cm rectangular chamber with the floor marked with a 16 × 16 grid. The test was conducted in a soundproof room. An animal was placed in the center of the chamber and left undisturbed for 5 min. The behaviors and locomotion tracking of the animal were recorded by an infrared digital video camera. The total distance traveled (cm), body center-point moving duration (s), highly mobile duration (s), and immobile (inactivity) duration (s) were analyzed using a commercially available software (EthoVision XT 8.5, Noldus, Netherlands).

#### Home cage locomotor activity test

Horizontal linear motion in the home cages was measured immediately after the open field test. The DSI activity sensor was activated with a magnet 30 min after the animals were placed back to their home cages. Spontaneous locomotor activity in the home cages were monitored continuously for 2 h without any external disturbances. The numbers of activity were recorded and analyzed using DSI software (Dataquest A.R.T system, DSI, USA).

### OXA immunohistochemistry

Animals were anesthetized with an overdose of sodium pentobarbital (100 mg/kg, i.p.) and then perfused transcardially with 100 ml chilled saline, followed by the perfusion with 500 ml 0.1 mol/l phosphate buffer (PB, pH 7.4) containing 4% paraformaldehyde. The brains were removed, post-fixed with 4% paraformaldehyde at 4°C for 1 h; a brain block including the hypothalamus was made and placed in 0.1 mol/l PB containing 30% sucrose overnight at 4°C. Then the blocks were cut into 20 μm-thick sections. One out of every 3 consecutive sections (2.2–3.4 mm caudal to the Bregma) where the OXA-labeled (OXA-LI) neurons were mainly located was selected for immunostaining.

The tissue sections were washed in 0.01 M phosphate-buffered solution (PBS, pH 7.4) and incubated in a mouse anti-OXA IgG (R&D Systems; 1:500) for 24 h at 4°C. After being washed in the PBS, the sections were incubated in a biotinylated horse anti-mouse IgG (Vector Laboratories; 1:200) for 4 h. OXA labeling was visualized using a ABC immunoperoxidase method. In brief, the samples were exposed to 1:100 streptavidin–horseradish peroxidase (HRP, Vector Laboratories, Burlingame, CA) for 4 h at 4°C. After being washed in PBS again, the sections were incubated in 0.05 M Tris-buffer (pH 7.6) containing 0.1% 3,3′-diaminobenzidine tetrahydrochloride (DAB; Sigma, St. Louis, MO) and 0.003% H_2_O_2_. Some control sections were processed without the primary antibody to rule out the non-specific immunostaining in these sections. Sections from comparable rostrocaudal levels of the hypothalamus (20 sections per animal) in each rat were chosen for cell counting. The number of the OXA-LI neurons was counted under a light microscope by a rater unaware of the experimental conditions. The photographs were taken with a digital camera.

### Drugs preparation

Sodium arsanilate (Sigma-Aldrich, St. Louis, USA) and IDPN (Fisher Scientific, Illkirch, France) were dissolved in 0.9% saline solution. JNJ7777120 (SelleckChem, Houston, USA) was prepared in 100% DMSO. SB-334867 (MedChem Express, Princeton, USA) was dissolved in the aCSF (in mM: 133.3 NaCl, 3.4 KCl, 1.3 CaCl_2_, 1.2 MgCl_2_, 0.6 NaH_2_PO_4_, 32.0 NaHCO_3_, and 3.4 glucose, with pH adjusted to 7.4).

### Data analysis

All statistical analyses were conducted using the SPSS v13.0 program. In Experiment 1, two-way ANOVA analysis was performed to examine the differences among the AVL, IVL or control groups. Fisher's LSD *post-hoc* test was used when a significant main effect or an interaction effect was obtained. In Experiment 2 and 3, one-way ANOVA analysis was performed to analyze the differences among groups followed by the Bonferroni *post-hoc* test when it is applicable. Data are expressed as the mean ± *SD*. The level of significance was set at 0.05.

## Results

### Behavior responses in the AVL and IVL animals

Table 1 shows the behavior responses after the AVL and IVL treatment in rats. Vestibular deficit analysis revealed significant effects of AVL [*F*_(1, 35)_ = 4748.80, *P* < 0.001] and time [*F*_(2, 35)_ = 45.49, *P* < 0.001] and an AVL × time interaction [*F*_(2, 35)_ = 45.49, *P* < 0.001]. Vestibular deficit scores were significantly increased at all of the time points in the AVL groups compared with the corresponding SHAM groups (*P* < 0.001). Significant IVL [*F*_(1, 35)_ = 4088.92, *P* < 0.001] and time [*F*_(2, 35)_ = 1067.50, *P* < 0.001] effects and an IVL × time interaction [*F*_(2, 35)_ = 1067.50, *P* < 0.001] were also observed. The IVL animals exhibited the vestibular deficit syndrome at 48 and 72 h after the injection of IDPN compared with the SAL controls (*P* < 0.001). The vestibular deficit syndrome was fully induced at 72 h in the AVL and IVL groups (Table 1). Neither the SHAM nor the SAL control animals showed any signs of the vestibular deficit symptom.

**Table 1:** **Effects of vestibular lesion (VL) on the vestibular deficit syndrome, home cage locomotor activity and exploratory behavior**.

	**Vestibular deficit score (%)**	**Home cage locomotor activity (counts/min)**	**Exploratory behaviors**			
			**Total distance traveled (cm)**	**Center-point moving (s)**	**Highly mobile duration (s)**	**Immobile duration (s)**
**24 H POST–VL**						
AVL	63.19 ± 6.67^***^	8.45 ± 0.96	40.51 ± 9.90^***^	64.20 ± 11.93^***^	1.63 ± 0.79	143.80 ± 21.27
SHAM	0.00 ± 0.00	9.44 ± 0.91	109.67 ± 14.76	105.33 ± 6.14	1.13 ± 0.78	124.43 ± 9.39
IVL	4.16 ± 3.72	9.51 ± 3.01	63.84 ± 7.86##	62.86 ± 13.08##	2.13 ± 1.47	150.03 ± 21.81##
SAL	0.00 ± 0.00	9.88 ± 2.43	144.45 ± 13.76	119.23 ± 3.28	0.86 ± 0.61	114.56 ± 7.82
**48 H POST–VL**						
AVL	84.72 ± 4.30^***^	14.78 ± 2.01^***^	176.56 ± 17.03	133.70 ± 15.65^*^	9.70 ± 3.97^**^	84.90 ± 21.44^**^
SHAM	0.00 ± 0.00	9.28 ± 1.92	121.15 ± 6.53	111.00 ± 6.79	1.77 ± 0.63	116.96 ± 8.58
IVL	52.77 ± 3.40###	11.81 ± 2.92	100.41 ± 17.61##	104.03 ± 13.70	3.90 ± 1.63	127.03 ± 12.04
SAL	0.00 ± 0.00	9.18 ± 3.09	124.02 ± 18.37	113.20 ± 9.06	1.46 ± 0.78	126.16 ± 15.05
**72 H POST–VL**						
AVL	87.50 ± 2.64^***^	22.81 ± 2.61^***^	307.74 ± 34.30^***^	162.70 ± 9.80^***^	16.66 ± 2.16^***^	37.93 ± 17.34^***^
SHAM	0.00 ± 0.00	10.83 ± 1.99	116.47 ± 26.49	112.00 ± 13.58	1.15 ± 0.37	121.43 ± 17.33
IVL	91.67 ± 2.63###	16.55 ± 2.02###	75.51 ± 19.78##	104.43 ± 23.38	2.68 ± 1.35#	120.90 ± 15.06
SAL	0.00 ± 0.00	10.87 ± 2.84	125.24 ± 23.13	109.73 ± 14.22	1.10 ± 0.62	116.16 ± 8.98

*AVL, Arsanilate-induced Vestibular Lesion; SHAM, Sham control; IVL, IDPN-Induced Vestibular Lesion; SAL, Saline control*.

* *P < 0.05*,

** P < 0.01, and

*** *P < 0.001 compared with corresponding SHAM group*.

# *P < 0.05*,

## P < 0.01, and

### *P < 0.001 compared with corresponding SAL group*.

Two-way ANOVA analysis revealed significant effects of AVL [*F*_(1, 35)_ = 54.593, *P* < 0.001] and time [*F*_(2, 35)_ = 68.417, *P* < 0.001] and an AVL × time interaction [*F*_(2, 35)_ = 30.242, *P* < 0.001] on locomotor activity in the home cages. *Post-hoc* analysis revealed that arsanilate significantly increased the number of activity at 48 h (*P* < 0.001) and 72 h (*P* < 0.001) in the AVL groups compared with the corresponding SHAM groups. There were also significant IVL [*F*_(1, 35)_ = 30.242, *P* = 0.012] and time [*F*_(2, 35)_ = 6.130, *P* = 0.006] effects on locomotor activity in the home cages. IDPN significantly increased the number of activity in the home cages at 72 h in the IVL group compared with the SAL group (*P* < 0.001, Table 1).

During the open field test, the AVL animals but not the SHAM controls showed increased exploratory behavior. Significant AVL [*F*_(1, 35)_ = 7.264, *P* < 0.001] and time [*F*_(2, 35)_ = 17.773, *P* < 0.05] effects and an AVL × time interaction [*F*_(2, 35)_ = 15.989, *P* < 0.001] on total distance traveled were observed. Total distance traveled was initially decreased at 24 h (*P* < 0.001), recovered back to the control level at 48 h and was significantly increased at 72 h (*P* < 0.001) in the AVL groups compared with the SHAM groups. Meanwhile, AVL [*F*_(1, 35)_ = 7.826, *P* < 0.01], time [*F*_(2, 35)_ = 66.764, *P* < 0.001] and AVL × time interaction [*F*_(2, 35)_ = 57.040, *P* < 0.0001] had significant effects on center point moving duration which was decreased at 24 h (*P* < 0.001) and increased at 48 h (*P* < 0.05) and 72 h (*P* < 0.001) in the AVL groups. A significant AVL effect was also observed in highly mobile duration [*F*_(1, 35)_ = 7.662, *P* < 0.01] and immobile duration [*F*_(1, 35)_ = 29.322, *P* < 0.001]. Highly mobile duration was increased at 48 h (*P* < 0.01) and 72 h (*P* < 0.001) after the injection of arsanilate, whereas immobile duration was decreased at these time points (*P* < 0.01 and *P* < 0.001) in the AVL groups compared with the SHAM groups.

Additionally, there was a significant IVL [*F*_(1, 35)_ = 33.624, *P* < 0.001] effect and an IVL × time interaction [*F*_(2, 35)_ = 4.204, *P* < 0.05] on total distance traveled which was significantly decreased in the IVL animals at all of the time points after the injection of IDPN compared with the SAL controls (*P* < 0.01). The IVL treatment also had significant effects on center-point moving [*F*_(2, 35)_ = 14.170, *P* < 0.001] as well as highly mobile duration [*F*_(2, 35)_ = 8.018, *P* < 0.01] and immobile duration [*F*_(2, 35)_ = 9.865, *P* < 0.005]. Compared with the SAL controls, the IVL animals showed a decrease in center-point moving duration and an increase in immobile duration at 24 h (*P* < 0.01) as well as an increase in highly mobile duration at 72 h (*P* < 0.05, Table 1). There were no significant effects of surgery or time on all of the variables in the SHAM or SAL groups during the open field test.

### Effects of AVL and IVLon OXA-labeling in the hypothalamus

**Figure 1:**
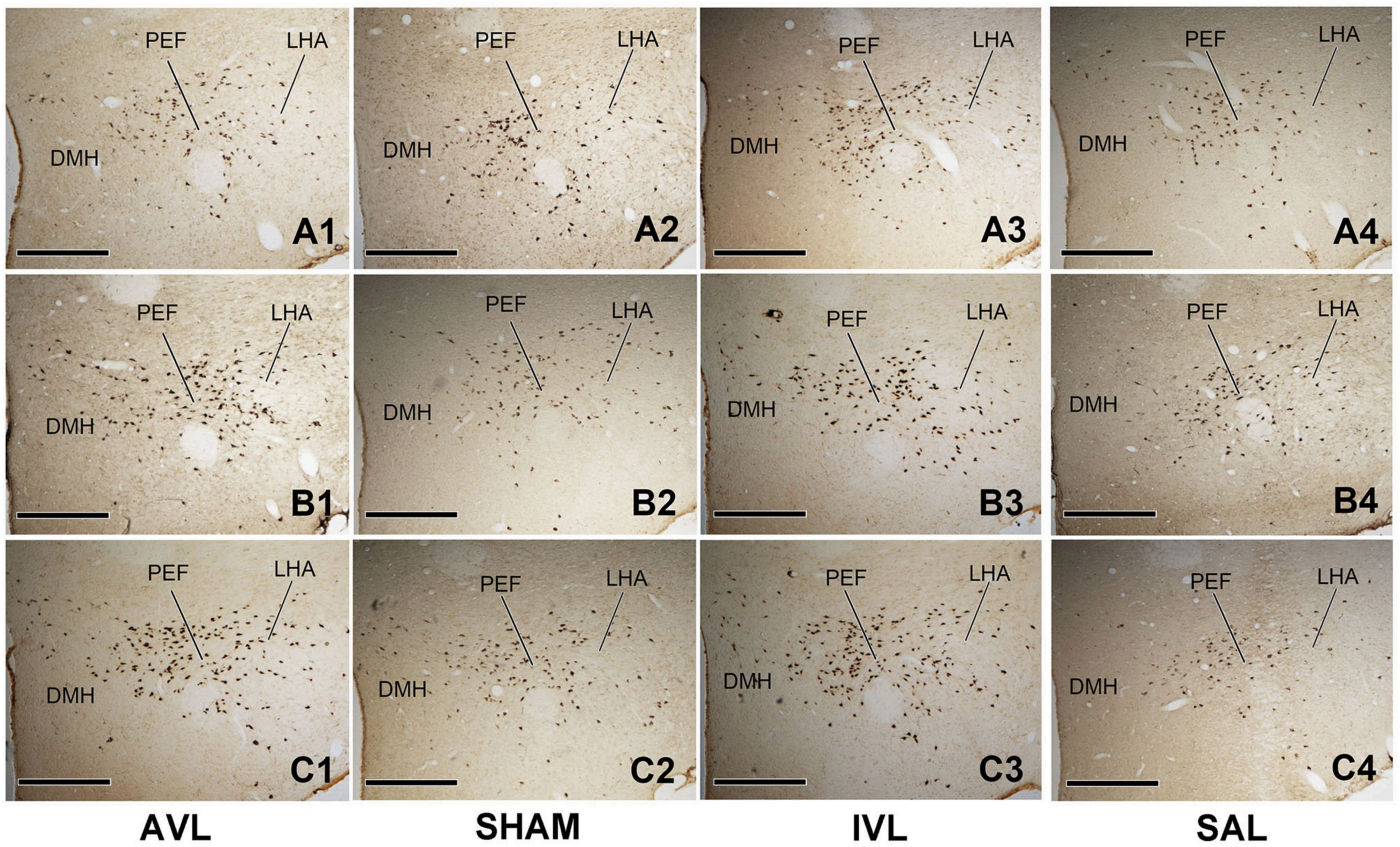
**Representative photomicrographs showing orexin-A-labeled (OXA-LI) neurons in rat hypothalamus (Bregma −2.6 ~ −3.0 mm) in the arsanilate-induced vestibular lesion (AVL), the sham control (SHAM), the IDPN-induced vestibular lesion (IVL), and the saline control (SAL) groups at 24 h (A1–A4), 48 h (B1–B4), or 72 h (C1–C4) after recovery from vestibular lesion or control treatment**. LHA, lateral hypothalamic area; PFA, perifornical area; DMH, dorsomedial hypothalamus. Bar = 400 μm.

Table 2 shows the numbers of the OXA-LI neurons in different regions of the rat hypothalamus (−2.2 ~ −2.6, −2.6 ~ −3.0, and −3.0 ~ −3.4 mm relative to the Bregma) after the AVL and IVL treatment. The densest concentration of the OXA-LI neurons was observed within Bregma −2.6 ~ –3.0 mm of the bilateral LHA, PFA, and DMH (Figure 1). The positive labeling was not observed in any control sections that were processed without the primary antibody. Two-way ANOVA analysis revealed significant AVL [*F*_(1, 35)_ = 9.264, *P* < 0.01] and time [*F*_(2, 35)_ = 6.371, *P* < 0.01] effects and an AVL × time interaction [*F*_(2, 35)_ = 6.763, *P* < 0.01], and a significant IVL [*F*_(2, 35)_ = 114.026, *P* < 0.001] effect and an IVL × time interaction [*F*_(2, 35)_ = 3.478, *P* < 0.05] on the number of the OXA-LI neurons within this region. *Post-hoc* analysis showed that the number of the OXA-LI neurons was significantly increased at 72 h in the AVL group compared with the SHAM group (*P* < 0.01; Figure 1C). Meanwhile, the IVL treatment increased the number of the OXA-LI neurons at all of the time points compared with the SAL control treatment (*P* < 0.01; Figures 1A,C). No significant differences were observed at 24 and 48 h between the AVL and SHAM groups (Figures 1A,B).

**Table 2:** **Effects of vestibular lesion (VL) on the number of orexin-A-labeled neurons in the hypothalamus**.

	**Bregma −2.2 ~ −2.6 mm**	**Bregma −2.6 ~ −3.0 mm**	**Bregma −3.0 ~ −3.4 mm**
**24 H POST–VL**			
AVL	45.00 ± 14.90	474.25 ± 20.79	250.00 ± 19.44
SHAM	51.00 ± 9.93	465.25 ± 30.12	213.00 ± 34.37
IVL	43.25 ± 9.11	609.00 ± 46.65##	380.50 ± 23.02
SAL	47.25 ± 7.13	459.75 ± 43.27	228.25 ± 31.85
**48 H POST–VL**			
AVL	35.50 ± 7.23	514.25 ± 28.86	298.50 ± 24.07
SHAM	42.37 ± 6.24	491.00 ± 27.60	247.50 ± 38.38
IVL	75.75 ± 16.74#	642.50 ± 23.29##	336.83 ± 26.22#
SAL	47.75 ± 12.09	455.75 ± 28.73	275.00 ± 33.64
**72 H POST–VL**			
AVL	69.37 ± 9.69^*^	579.50 ± 29.16^**^	343.00 ± 40.16^**^
SHAM	43.00 ± 8.29	467.25 ± 29.65	235.00 ± 32.54
IVL	80.25 ± 14.08##	603.50 ± 38.21##	336.25 ± 30.27##
SAL	37.00 ± 10.74	497.73 ± 31.87	253.50 ± 27.01

*AVL, Arsanilate-induced Vestibular Lesion; SHAM, Sham control; IVL, IDPN-Induced Vestibular Lesion; SAL, Saline Control*.

* *P < 0.05*,

** P < 0.01 compared with corresponding SHAM group;

# *P < 0.05*,

## *P < 0.01 compared with corresponding SAL group*.

Two-way ANOVA analysis also found a significant AVL [*F*_(1, 35)_ = 21.296, *P* < 0.001] effect and an AVL × time interaction [*F*_(2, 35)_ = 12.179, *P* < 0.001]; significant IVL [*F*_(2, 35)_ = 48.506, *P* < 0.001] and time [*F*_(1, 35)_ = 10.789, *P* < 0.001] effects and an IVL × time interaction [*F*_(2, 35)_ = 14.019, *P* < 0.001] on the number of the OXA-LI neurons within Bregma −2.2 ~ −2.6 mm. Significant AVL [*F*_(2, 35)_ = 20.330, *P* < 0.01] and time [*F*_(1, 35)_ = 7.709, *P* < 0.01] effects and a significant IVL [*F*_(2, 35)_ = 25.141, *P* < 0.001] effect on the number of OXA-LI neurons were also observed within Bregma −3.0 ~ −3.4 mm. The numbers of the OXA-LI neurons in these regions were significantly increased at 72 h in the AVL group (*P* < 0.05, Figure 1C) and at 48 h (*P* < 0.05, Figure 1B) and 72 h (*P* < 0.01, Figure 1C) in the IVL groups compared with the corresponding control groups.

### Effects of JNJ7777120 on the behavior responses in the AVL and IVL animals

Table 3 shows the effects of the i.p. injection of the H_4_R inhibitor JNJ7777120 on the behavior responses at 72 h after the AVL and IVL treatment. Compared with the corresponding SHAM-Veh and SAL-Veh controls, the AVL-Veh and IVL-Veh animals exhibited the vestibular deficit syndrome and hyperactivity in the home cages (*P* < 0.001 or *P* < 0.01). Vestibular deficit score was significantly decreased after JNJ7777120 administration in the AVL and IVL groups but was still higher than in the SHAM-Veh or SAL-Veh groups (*P* < 0.001). JNJ7777120 also inhibited hyperactivity in the home cages in the AVL and IVL groups compared with corresponding Veh groups (*P* < 0.001, Table 3).

**Table 3:** **Effects of the histamine H_**4**_ receptor antagonist JNJ7777120 on the vestibular deficit syndrome, home cage locomotor activity and exploratory behavior at 72 h post-vestibular lesion (VL)**.

	**Vestibular deficit score (%)**	**Home cage locomotor activity (counts/min)**	**Exploratory behavior**			
			**Total distance traveled (cm)**	**Center-point moving (s)**	**Highly mobile duration (s)**	**Immobile duration (s)**
**AVL EXPERIMENT**						
AVL-Veh	86.81 ± 4.43^***^	23.77 ± 4.71^***^	361.57 ± 35.00^***^	138.26 ± 29.14^*^	13.80 ± 2.64^***^	59.46 ± 35.86^***^
AVL-JNJ	63.19 ± 5.63^***^###	13.25 ± 2.63###	53.44 ± 36.57^**^###	62.09 ± 36.30^*^###	1.70 ± 1.01###	160.45 ± 19.18^***^###
SHAM-Veh	0.00 ± 0.00	10.67 ± 2.22	109.84 ± 10.48	104.54 ± 12.48	1.80 ± 1.50	125.28 ± 6.26
SHAM-JNJ	0.00 ± 0.00	9.75 ± 2.63	99.12 ± 12.37	93.85 ± 15.32	0.56 ± 0.35	131.25 ± 4.67
**IVL EXPERIMENT**						
IVL-Veh	83.33 ± 5.55^***^	18.09 ± 2.06^**^	85.19 ± 18.29^*^	123.83 ± 22.45	3.86 ± 1.60^*^	123.23 ± 21.83
IVL-JNJ	56.94 ± 7.71^***^###	7.42 ± 1.53###	50.01 ± 14.02^***^#	68.52 ± 28.40^**^##	1.13 ± 1.03#	153.86 ± 20.10^**^#
SAL-Veh	0.00 ± 0.00	10.52 ± 3.72	115.15 ± 25.02	103.75 ± 13.24	0.47 ± 0.28	117.68 ± 16.40
SAL-JNJ	0.00 ± 0.00	8.57 ± 1.99	97.43 ± 15.31	107.20 ± 11.26	0.44 ± 0.32	122.86 ± 12.25

*AVL, Arsanilate-induced Vestibular Lesion; SHAM, Sham control; IVL, IDPN-Induced Vestibular Lesion; SAL, Saline control Veh, vehicle solution; JNJ, JNJ7777120*.

* *P < 0.05*,

** P < 0.01, and

*** *P < 0.001 compared with corresponding SHAM-Veh or SAL-Veh group*.

# *P < 0.05*,

## P < 0.01, and

### *P < 0.001 compared with corresponding AVL-Veh or IVL-Veh group*.

During the open field test, the AVL-Veh treatment significantly increased total distance traveled (*P* < 0.001), center-point movement duration (*P* < 0.05), and highly mobile duration (*P* < 0.001) and decreased immobile duration (*P* < 0.001), whereas the IVL-Veh treatment significantly increased highly mobile duration (*P* < 0.05) and deceased total distance traveled (*P* < 0.05) compared with the corresponding SHAM-Veh and SAL-Veh treatments (Table 3). JNJ7777120 significantly reduced total distance traveled, center-point moving duration and highly mobile duration and increased immobile duration in the AVL animals compared to the vehicle controls (*P* < 0.001). Total distance traveled (*P* < 0.01) and center-point moving duration (*P* < 0.05) were significantly decreased, while immobile duration was significantly increased (*P* < 0.001) in AVL-JNJ group compared with SHAM-Veh group. JNJ-7777120 also significantly reduced total distance traveled (*P* < 0.05), center-point movement duration (*P* < 0.01) and highly mobile duration (*P* < 0.05), and significantly increased immobile duration (*P* < 0.05) in the IVL animals compared to the vehicle controls. JNJ7777120 also significantly decreased total distance traveled (*P* < 0.001) and center-point movement duration (*P* < 0.01) and increased immobile duration (*P* < 0.01) in the IVL-JNJ group compared with the SAL-Veh group. No changes in any of the open field variables were observed after the i.p. injection of JNJ7777120 in the SHAM or SAL control animals (Table 3).

### Effects of SB334867 on behavior responses in the AVL and IVL animals

**Figure 2:**
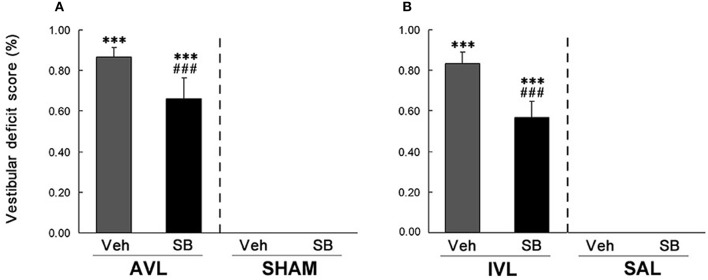
**Effects of the orexin receptor type 1 (OX_**1**_R) antagonist SB334867 on the vestibular deficit syndrome after vestibular lesions induced by arsanilate (A) and IDPN (B) in rats**. AVL, arsanilate**-**induced vestibular lesion; SHAM, sham control; IVL, IDPN**-**induced vestibular lesion; SAL, saline control; Veh, vehicle solution; SB, SB334867. Data are represented as mean ± SEM. ^***^P < 0.001 compared with SHAM or SAL, vehicle control group; ^###^*P* < 0.001 compared with AVL or IVL vehicle control group.

**Figure 3:**
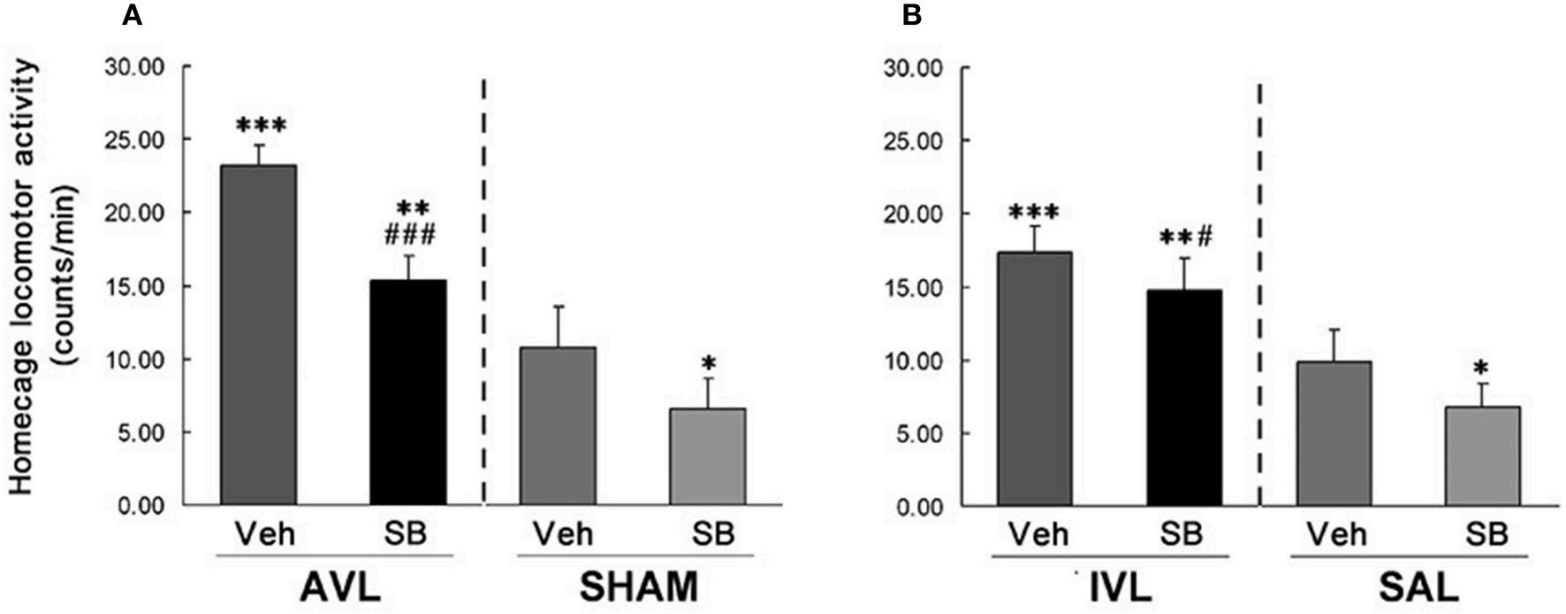
**Effects of the orexin receptor type 1 (OX_**1**_R) antagonist SB334867 on locomotor activity in the home cages after vestibular lesions induced by arsanilate (A) and IDPN (B) in rats**. AVL, arsanilate**-**induced vestibular lesion; SHAM, sham control; IVL, IDPN**-**induced vestibular lesion; SAL, saline control; Veh, vehicle solution; SB, SB334867. Data are represented as mean ± SEM. ^*^P < 0.05, ^**^P < 0.01, ^***^P < 0.001 compared with SHAM or SAL, vehicle control group; ^#^*P* < 0.05, ^###^*P* < 0.001 compared with AVL or IVL vehicle control group.

In this experiment, vestibular deficit syndrome and hyperactivity in the home cages were successfully induced in the AVL-Veh and IVL-Veh animals compared with the corresponding SHAM-Veh and SAL-Veh controls (*P* < 0.001, Figures 2, 3). In the AVL-SB and IVL-SB groups, the i.c.v injection of the OX_1_R antagonist SB334867 significantly reduced vestibular deficit scores compared with the corresponding Veh groups (*P* < 0.001) and the vestibular deficit scores were still higher than in the SHAM-Veh and SAL-Veh groups (*P* < 0.001, Figures 2A,B). SB334867 also attenuated the hyperactivity induced by the AVL and IVL in the home cages (*P* < 0.001 and *P* < 0.05) compared with the vehicle controls., The amounts of the locomotor activity were still higher in the AVL-SB and IVL-SB groups than in the SHAM-Veh and SAL-Veh groups (*P* < 0.01, Figures 3A,B). In addition, SB334867 also reduced the locomotor activity in the home cages in the SHAM and SAL animals compared with corresponding Veh controls (*P* < 0.05, Figures 3A,B).

**Figure 4:**
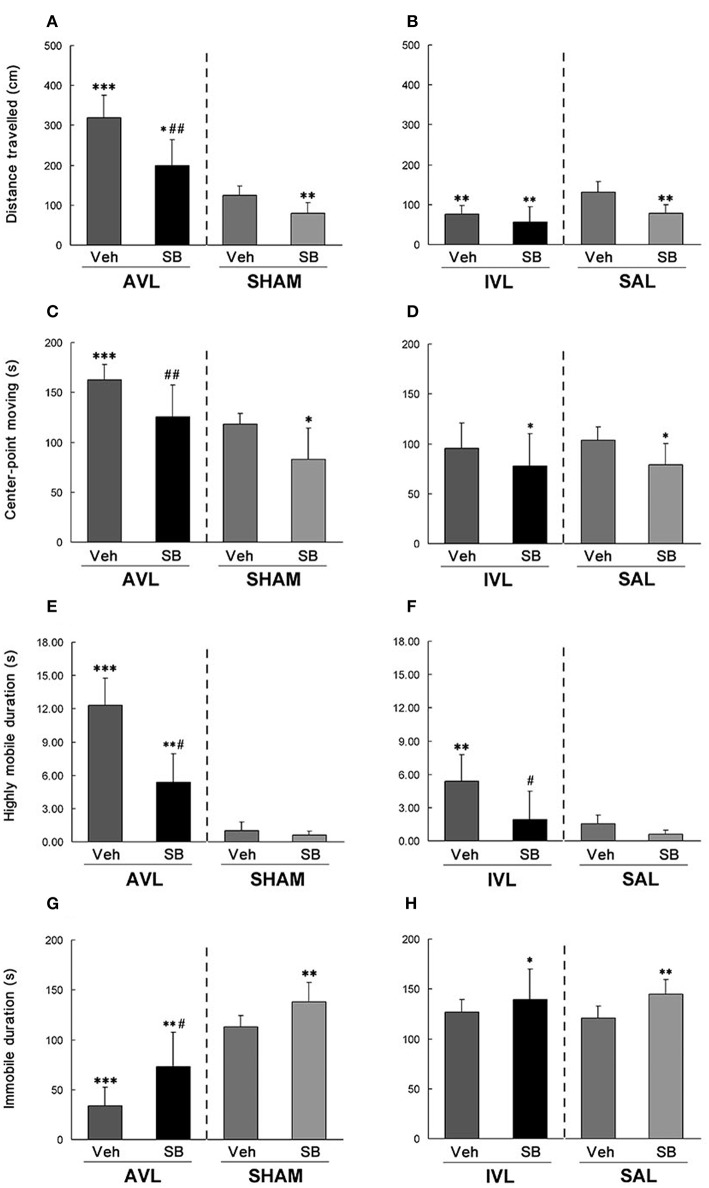
**Effects of the orexin receptor type 1 (OX_**1**_R) antagonist SB334867 on exploratory behavior in the open field after vestibular lesions induced by arsanilate and IDPN in rats**. Total distance traveled **(A,B)**, body center-point moving duration **(C,D)**, highly mobile duration **(E,F)** and immobile duration **(G,H)** were analyzed; AVL, arsanilate**-**induced vestibular lesion; SHAM, sham control; IVL, IDPN**-**induced vestibular lesion; SAL, saline control; Veh, vehicle solution; SB, SB334867. Data are represented as mean ± SEM. ^*^P < 0.05, ^**^P < 0.01, ^***^P < 0.001 compared with SHAM or SAL vehicle control group; ^#^*P* < 0.05, ^*##*^*P* < 0.01 compared with AVL or IVL vehicle control group.

During the open field test, AVL-Veh animals showed significant increases in total distance traveled (*P* < 0.001, Figure 4A), center-point moving duration (*P* < 0.001, Figure 4C) and highly mobile duration (*P* < 0.001, Figure 4E) and a decrease in immobile duration (*P* < 0.001, Figure 4G) compared with the SHAM-Veh controls. The IVL-Veh animals exhibited a significant increase in highly mobile duration (*P* < 0.01, Figure 4F) and a decrease in total distance traveled (*P* < 0.01, Figure 4B) compared with the SAL-Veh controls. SB334867 significantly reduced total distance traveled (*P* < 0.01, Figure 4A), center-point moving duration (*P* < 0.01, Figure 4C) and highly mobile duration (*P* < 0.05, Figure 4E), and increased immobile duration (*P* < 0.05, Figure 4G) in the AVL-SB animals compared with AVL-Veh controls. Total distance traveled (*P* < 0.05, Figure 4A) and highly mobile duration (*P* < 0.01, Figure 4E) were increased and immobile duration was decreased (*P* < 0.01, Figure 4G) in the AVL-SB group compared with the SHAM-Veh group. In the IVL animals, SB334867 reduced highly mobile duration compared to the vehicle (*P* < 0.05, Figure 4F). SB334867 also decreased total distance traveled (*P* < 0.01, Figure 4B) and center-point moving duration (*P* < 0.05, Figure 4D) and increased immobile duration (*P* < 0.05, Figure 4H) compared with SAL-Veh controls. In the SHAM and SAL animals, SB334867 reduced total distance traveled (*P* < 0.01, Figures 4A,B) and center-point moving duration (*P* < 0.05, Figures 4C,D) and increased immobile duration (*P* < 0.01, Figures 4G,H) but did not affect highly mobile duration (*P* > 0.05, Figures 4E,F) compared with corresponding Veh controls.

## Discussion

In the present study, arsanilate and IDPN successfully elicited the vestibular deficit syndrome and hyperactivity in the home cages in rats. These results were consistent with previous observations which showed that both arsanilate and IDPN can damage the vestibular endorgans in rodents (Boadas-Vaello et al., 2005; Vignaux et al., 2012). Nevertheless, the exploratory behavior in an open field was increased by arsanilate but decreased by IDPN. Such discrepancy might possibly be due to the differences in the toxicity of these two chemicals. For example, the intra-tympanic injection of arsanilate causes extensive damages in the vestibular epithelia while the systemic application of IDPN induces an inhomogeneous degeneration (Llorens and Demômes, 1996; Boadas-Vaello et al., 2005; Vignaux et al., 2012; Martin et al., 2015). IDPN can also induce some neuropathological effects such as axonal swellings and neurofilamentous accumulation in the central nervous system and cause reactive gliosis in the retina and olfactory bulbs and the reversible opacification in the cornea (Balbuena and Llorens, 2001; Boadas-Vaello et al., 2005; Khan et al., 2009). In addition, we also showed that the H_4_R antagonist significantly alleviated the vestibular deficit syndrome and completely abolished hyperactivity in the home cages in the arsanilate- and IDPN-treated animals. Blockade of the H_4_R also significantly reduced exploratory behavior in an open field in these animals. Although the H_4_R is expressed in the cortex, the cerebellum and the spinal cord (Strakhova et al., 2009), the intracerebroventricular administration of H_4_R agonist failed to alter the spontaneous mobility and the exploratory activity in mice (Galeotti et al., 2013). This indicates that the vestibular neurons but not the central targets were influenced by the blockade of the H_4_R (Wersinger et al., 2013). These evidences also suggest that the vestibular lesions were the most likely responsible for the behavior abnormalities that were observed in this study.

Previous studies indicated that orexin expression in the LHA neurons can be stimulated by homeostatic perturbations that were induced by altered sleep and metabolism, exposure to drugs, or other noxious insults (Machaalani et al., 2013). Functional studies have demonstrated that the vestibular system is involved in regulating biological circadian, autonomic responses and energy metabolism in the context of vestibular pathology and balance disorder (Fuller et al., 2002). For example, the activation of the vestibular system can disrupt energy homeostasis via enhancing the thermolysis and suppressing the resting energy expenditure during motion sickness in humans (Cheung et al., 2011; Wang et al., 2016). Disrupted daily rhythms of the body temperature and physical activity were observed in animals with vestibular loss caused by genetic mutation or arsanilate injection (Fuller et al., 2002; Martin et al., 2015). In the present study, we revealed that the OXA expression in the hypothalamus was increased in the arsanilate-treated animals at the time when the vestibular deficit syndrome and locomotor hyperactivity were fully produced. As for the orexin system also play important roles in circadian regulation, energy homeostasis, and autonomic responses (Boss and Roch, 2015), our results suggest that the vestibular disorder-induced homeostatic disturbances might be associated with alterations in orexin activity. However, whether the vestibular system can directly regulate the activity of the orexin system is not clarified in this study and needs further investigations.

In the IDPN-treated animals, the orexin expression was increased before the vestibular deficit syndrome was significantly induced. It seems that IDPN may influence the orexin activity through different mechanisms compared with arsanilate which only induces damages restricted to the vestibular sensory organs (Vignaux et al., 2012). One explanation may involve the direct effects of IDPN on monoamines contents in the brain. IDPN primarily affects the 5-hydroxytryptamine (5-HT) containing neurons, leading to a widely decrease in the 5-HT and 5-hydroxyindoleacetic acid levels in all of the brain regions (Langlais et al., 1975; Wakata et al., 2000). IDPN also can reduce dopamine and its metabolites' levels through depressing dopamine metabolic turnover (Wakata et al., 2000). The deceases in monoamine levels are likely to stimulate the orexin system via deactivation of the 5-HT_1*A*_ and dopamine D_2_ receptor sites in the LHA (Muraki et al., 2004; Bubser et al., 2005). Another possible mechanism may involve oxidative stress induced by IDPN in the brain through the excessive generation of the oxygen-derived free radicals via the depletion of glutathione (Tariq et al., 2002; Ahmad Khan et al., 2004; Stefanescu and Ciobica, 2012). Free radical scavenger that inhibits the process of hydroxyl radical formation is effective against the vestibular deficit syndrome induced by IDPN (Nomoto, 2004). Recent studies showed that OXA has anti-oxidant and anti-apoptotic effects against neuronal damage (Esmaeili-Mahani et al., 2013). Therefore, we speculate that the increase in orexin expression might be a result of IDPN-induced oxidative stress in the central nervous system.

In the current study, the OX_1_R antagonist SB334867 significantly alleviated the vestibular deficit syndrome and locomotor hyperactivity in the home cages in the AVL and IVL animals. These results suggested that the elevated OXA activity might play a role in vestibular loss-induced locomotor deficits and hyperkinesia. Previous studies suggest that locomotor hyperactivity and vestibular deficit syndrome might be due to the altered dopaminergic activity via vestibular–basal ganglia connection in vestibular-deficient animals (Stiles and Smith, 2015). It is noteworthy that the basal ganglia subregions such as the striatum, the subthalamic nucleus and the substantia nigra pars compacta are also densely innervated by the orexinergic fibers and express the orexinergic receptors (Hu et al., 2015). Microinjection of orexin into the substantia nigra pars compacta apparently increased the movement time in rats (Kotz et al., 2006). However, the orexin neurons also project widely to the centers of the ascending activating system including the laterodorsal tegmental, the pedunculopontine tegmental, the basal forebrain, the locus coeruleus, the dorsal raphe and the tuberomamillary nucleus (Sinton, 2011). Pharmacological and optogenetic studies have demonstrated that these connections not only participate in regulating sleep/wake transition, but also play a role in modulating arousal and locomotor activity (Hagan et al., 1999; Adamantidis et al., 2007). Furthermore, the orexin system can also regulate the resting and active motor thresholds via the connections with the motor cortex (Peyron et al., 1998; Oliviero et al., 2005). Orexin knockout mice exhibited a significant decrease in voluntary motor activity compared with the wild type animals (Hara et al., 2001). These evidences suggest that vestibular loss-induced spontaneous or voluntary locomotor hyperactivity might involve multiple motor control structures that receive the orexinergic innervations. Nevertheless, the current study also revealed that SB334867 only attenuated circling, retropulsion and abnormal head movements while the contact inhibition of the righting reflex, the tail-hanging reflex and the air-righting reflex still remained abnormal in the VL animals. Functional and anatomical studies confirmed that the vestibular nuclei receive the projections from the cortex, the cerebellum and some brainstem structures in addition to the vestibular nerve inputs (Cullen, 2012). Clinical investigations in patients showed that the loss of vestibular-ocular reflexes and vestibulo-spinal reflexes resulted in oscillopsia and gait ataxia, respectively (Mamoto et al., 2002). In addition, bilateral microinjection of SB334867 into the lateral vestibular nucleus did not affect rat motor performance in either the horizontal balance beam or the negative geotaxis test (Zhang et al., 2011). These evidences suggest that the orexin system may not regulate balance and postural control which involves the multimodal interactions and multiple vestibular reflex pathways.

Our study also showed that the OX_1_R antagonist attenuated vestibular loss-induced exploratory behavior in a new environment. This result is in agreement with a previous observation showing that activation of the orexin system increased exploration and arousal in a novel environment in rats. (Heydendael et al., 2014). Nevertheless, microinjection of SB334867 into bilateral vestibular nucleus did not affect the exploratory activity in an open field, indicating that orexin regulates these behaviors via acting on other brain structures (Zhang et al., 2011). In addition, it was reported that the orexin neurons were activated by a fear-conditioned cue, but not by restraint stress (Furlong et al., 2009). The orexin receptor blockade significantly reduced the cardiovascular and exploratory responses induced by fear but not by cold exposure (Furlong et al., 2009). These evidences strongly suggest that orexin neurons can be activated by psychological stressors but not by physical stressors. In the present study, in order to minimize pain and physical stress that were induced by the vestibular lesions, we set sham operation and saline control groups in the AVL and IVL experiment, respectively, and used antibiotics and analgesics after the intra-tympanic injection of arsanilate. Given that the vestibular lesions in the acute phase significantly induced hyperactivity but not led to hypoactivity that is commonly seen in psychologically stressed animals, we speculate that acute stress might not be the activator of the orexin neurons. Interestingly, clinical investigations described that patients with the vestibular disorders, such as migrainous vertigo and Menière disease, have high rates of coexistence of psychiatric disorders especially depression/anxiety (Yuan et al., 2015). Vestibular stimuli exceeding the capacity to integrate multimodal information, not only generates the typical motion sickness-related physiological responses, but also contributes to anxiety and fear under specific circumstances (Coelho and Balaban, 2015). A recent study found that vestibular stimulation relieved depression and anxiety and improved sleep quality and autonomic functions in college students that were exposed to the competitive environments in college (Kumar et al., 2016). These results suggest that the vestibular system might play a role in regulating motivated behaviors and psychological responses. However, whether orexin is also involved in modulating psychological responses in animals and humans with vestibular dysfunction is still unclear and merits further investigation.

In summary, vestibular deficit syndrome and locomotor hyperactivity were fully induced in rats that received the AVL and IVL treatment. The AVL animals also exhibited increased exploratory behavior in an open field. The H_4_R inhibitor attenuated the vestibular deficit syndrome and eliminated locomotor and exploratory hyperactivity in both AVL and IVL animals, indicating that these behavior responses were mainly induced by vestibular lesion. The novel findings of the present study were that the AVL and IVL treatment significantly increased orexin expression in the hypothalamus. The OX_1_R inhibitor significantly attenuated the vestibular deficit syndrome and locomotor hyperactivity in both AVL and IVL animals, and decreased exploratory behavior in the AVL animals compared with the vehicle controls. These results suggest that the vestibular system may potentially regulate the activity of the orexin system which might contribute to the acute locomotor abnormalities that can be induced vestibular damage or dysfunctions.

## Author contributions

JL was responsible for animal breeding. LP and RQ built the vestibular loss rat model. LP, RQ, JW, WZ, and JL performed behavioral and pharmacological experiment. LP, RQ, and WZ performed immunohistochemistry experiment. LP, RQ, JW, and YC were responsible for the design of the work. LP, JW, and WZ were responsible for data analysis. YC, LP, and RQ were responsible for writing the manuscript. All authors read and approved the final manuscript.

### Conflict of interest statement

The authors declare that the research was conducted in the absence of any commercial or financial relationships that could be construed as a potential conflict of interest. The reviewer TH and handling Editor declared their shared affiliation, and the handling Editor states that the process nevertheless met the standards of a fair and objective review.
